# Use of a Low-Cost, Chest-Mounted Accelerometer to Evaluate Transfer Skills of Wheelchair Users During Everyday Activities: Observational Study

**DOI:** 10.2196/11748

**Published:** 2018-12-20

**Authors:** Giulia Barbareschi, Catherine Holloway, Nadia Bianchi-Berthouze, Sharon Sonenblum, Stephen Sprigle

**Affiliations:** 1 University College London Interaction Centre London United Kingdom; 2 Rehabilitation Engineering and Applied Research Laboratory Georgia Institute of Technology Atlanta, GA United States

**Keywords:** wheelchair transfers, movement evaluation, machine learning, activity monitoring, accelerometer

## Abstract

**Background:**

Transfers are an important skill for many wheelchair users (WU). However, they have also been related to the risk of falling or developing upper limb injuries. Transfer abilities are usually evaluated in clinical settings or biomechanics laboratories, and these methods of assessment are poorly suited to evaluation in real and unconstrained world settings where transfers take place.

**Objective:**

The objective of this paper is to test the feasibility of a system based on a wearable low-cost sensor to monitor transfer skills in real-world settings.

**Methods:**

We collected data from 9 WU wearing triaxial accelerometer on their chest while performing transfers to and from car seats and home furniture. We then extracted significant features from accelerometer data based on biomechanical considerations and previous relevant literature and used machine learning algorithms to evaluate the performance of wheelchair transfers and detect their occurrence from a continuous time series of data.

**Results:**

Results show a good predictive accuracy of support vector machine classifiers when determining the use of head-hip relationship (75.9%) and smoothness of landing (79.6%) when the starting and ending of the transfer are known. Automatic transfer detection reaches performances that are similar to state of the art in this context (multinomial logistic regression accuracy 87.8%). However, we achieve these results using only a single sensor and collecting data in a more ecological manner.

**Conclusions:**

The use of a single chest-placed accelerometer shows good predictive accuracy for algorithms applied independently to both transfer evaluation and monitoring. This points to the opportunity for designing ubiquitous-technology based personalized skill development interventions for WU. However, monitoring transfers still require the use of external inputs or extra sensors to identify the start and end of the transfer, which is needed to perform an accurate evaluation.

## Introduction

Globally, there are over 70 million wheelchair users (WU), and there is a growing need for wheelchairs to fill the mobility gap for people who are unable, or struggle, to walk [1]. This is a trend we can expect to continue as the population ages, and more people live longer with conditions that affect their ability to walk. Wheelchairs can be manual, electric or have “power assist,” which gives additional power with each push. Regardless of the type of wheelchair being used, the user needs to get into and out of the wheelchair. This process is called transferring.

Transfers are necessary for many daily activities and happen on average between 14 and 18 times a day [2,3]. Transfers occur between the wheelchair and other surfaces, and they are affected by a variety of factors such as height and stability of the surfaces and space available around them. Depending on the environment and the characteristics of the person, each transfer will have its challenges [4,5].

Learning how to transfer correctly is a critical skill for WU. In order to maintain this independence WU must preserve the functioning in their upper limbs. However, due to the exceptionally high loads, and the repetitive nature of wheelchair transfers, WU frequently suffer from pain in the shoulders and wrists [6]. This pain is caused by musculoskeletal injuries, which can prevent people from using their wheelchair independently.

Wheelchair skills training helps to prevent such injuries by teaching WU correct techniques for everyday activities such as pushing over a variety of surfaces and transferring onto and from many surfaces. Clinicians mostly deliver wheelchair skills training [7] within rehabilitation clinics, but it can also be provided through charities that offer peer-to-peer training, or even remotely via online courses [8]. Regardless of how the training is provided, patients still need to rely on clinicians to evaluate their transfers, and this evaluation generally takes place in the clinic.

Indeed, there is no routine way for wheelchair transfers to be monitored remotely in everyday life settings. Furthermore, the provision of wheelchair skills training is not universal and can depend on geography (eg, more prevalent in more affluent countries), medical diagnosis (eg, spinal injury rehabilitation programs generally integrate wheelchair skills training whereas, for other conditions such as stroke, rehabilitation units might not), and funding [9]. The Web-based e-learning platform piloted by Worobey et al [8] shows the potential to improve the availability of transfer training through massive open online courses, but would benefit from a method for home/self-monitoring for WU that would ensure they did not need to depend on a clinician.

Wearable technologies offer the opportunity to provide monitoring and feedback to WU during their daily lives, particularly on activities and techniques which are known to cause injury. Research in this area has focused on automatically detecting different types of activities from one another (eg, resting, pushing the wheelchair, performing household activities) [10,11]. Most authors have focused on the use of a wrist-worn sensor for activity monitoring [12-14]. A few have linked energy expenditure to accelerometer data [14,15], and 1 has investigated the quality of pushes, identifying a good style of pushing from a poor one [12]. Very little attention has been dedicated to transfers.

There has only been 1 study which has evaluated the accuracy of classification algorithms for detecting the occurrence of wheelchair transfers, alongside other activities [11]. The researchers used 4 accelerometers located at the wrists, chest, and waist [11]. The experiment was highly successful, and transfer recognition reached 100% accuracy for both quadratic discriminant analysis (QDA) and support vector machine (SVM). However, the study consisted of a highly controlled experimental set-up and involved the performance of consecutive transfers for a set period, reducing movement differences between repetitions of the same activity. Also, the transfers were only executed between 2 surfaces of the same height (2 wheelchairs) rather than between different types of surfaces and different environmental real-life contexts. Therefore, it is not clear if the results generalize to real-life settings. Finally, despite the use of 4 sensors, only in-depth analysis of the contribution of the wrist-worn sensors is reported and it is not clear to what extent the other sensors contribute to the recognition. This is particularly critical given that trunk-worn sensors are, for example, useful for evaluating aspects of transfer quality [16] and WU do not always appreciate wrist-worn sensors as they can interfere with the wheel during pushing [17].

The primary aim of this study was to develop a strategy to enable the use of a single low-cost wearable sensor to evaluate the quality of wheelchair transfers across 3 common transfer scenarios. Body-worn sensors are often used to detect movement (ie, recognition). However, they are rarely used to evaluate the quality of body movement [18]. This is especially true for rehabilitation purposes, as the system needs to be able to capture clinical expertise in evaluating the movement. The secondary aim was to adapt current methods for the detection of wheelchair transfer occurrences through the same sensor in more ecologic settings with the long-term aims of continuously monitoring transfer skills.

## Methods

### Recruitment

The study was approved by the Internal Review Board at the Georgia Institute of Technology, United States. Calls for participants were made via a laboratory database, recruitment flyers, social media, and relevant online forums. Interested subjects were screened against the following criteria: (1) between 18-65 years of age, (2) use of a wheelchair as primary means of mobility for at least six months, and (3) ability to perform wheelchair transfers independently. Participants were excluded if they (1) were able to fully stand up when transferring, (2) reported the use of a transfer board when transferring, (3) were currently admitted to a hospital or a rehabilitation facility, and (4) reported having upper extremity pain or any medical condition that was likely to be exacerbated through the study protocol (eg, angina, exercise-induced asthma, uncontrolled hypertension).

### Equipment

In this study, we consider the use of 1 accelerometer placed on the chest of the user to measure g-force acceleration. The accelerometer was secured to the upper third of the sternum of the participants using double-sided tape. The chest was chosen as it is the part of the body which dictates a good transfer (eg, turning the trunk to align a good head-hip relationship) and is helpful in detecting the start (eg, forward lean of the trunk) and end points of the transfer (eg, controlled descent). Also, the trunk is in motion throughout the wheelchair transfer cycle, whereas the arms are often stationary during key moments in the transfer [19,20]. Finally, the upper third of the sternum was chosen as the location as it guarantees good stability measurements [21]. The use of a single accelerometer was preferred to a multi-sensor system, as future applications for long term-monitoring will need to be as unobtrusive as possible in order to maximize the ease of use.

**Figure 1:**
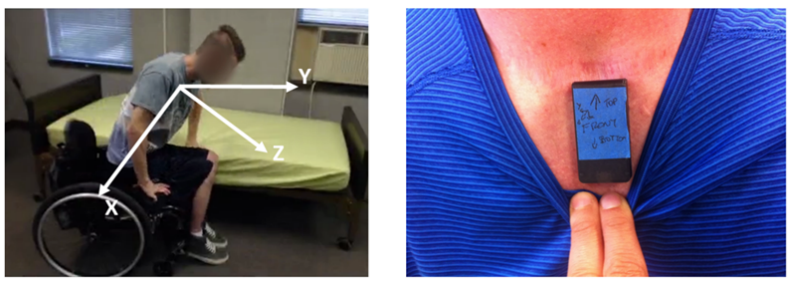
The orientation of the accelerometer’s axes relative to the body during wheelchair transfers and its position on the participant’s sternum.

Trunk accelerations were recorded using a single wireless 3-axis accelerometer (range ±16g, resolution 16-bit, Gulf Coast Data Concepts, MS) sampling at 25Hz. The directions of the acceleration (see Figure 1) were measured in respect to the individual body axes (+ up – down; Y + left – right; Z + front – back). The accelerometer data were filtered using an 8^th^ order low pass Butterworth filter with a cut off frequency of 10Hz to reduce noise. Two video cameras were used to record participants’ transfers, to label the recorded data for transferring quality performance and to determine exact seat-off (start) and landing time (end). Data processing was carried out on MATLAB R2015b, and the accuracy of various classifiers was calculated using WEKA 3.8 data mining suite.

### Data Collection

**Figure 2:**
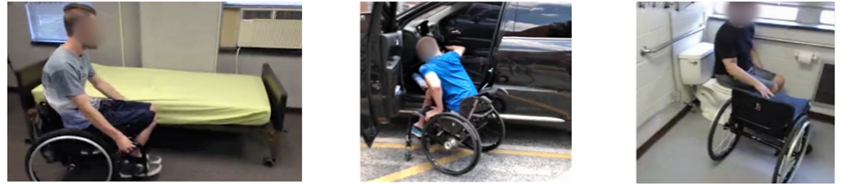
Bed, car, and toilet transfer scenarios.

For the data collection, a series of ecologically valid scenarios (see Figure 2) was used consisting of 3 common daily transfers: wheelchair-bed, wheelchair-toilet, and wheelchair-car. The first 2 represent necessary daily activities while car transfers are the most crucial skill for personal independence and social/working life [22]. The wheelchair-bed scenario was recreated in the research facility, and a real accessible bathroom in the building was used for the wheelchair-toilet scenario. The participant’s vehicle was used for the wheelchair-car scenario, as all participants reported ownership of a car.

Participants were asked to perform 2 return transfers (ie, to and from the wheelchair) for each scenario using their own wheelchair. Participants were asked to complete the transfers as they normally would in their everyday lives. The order of the 3 scenarios was randomized across participants. Also, between each transfer, the person was asked to move around the room to ensure variability between transfer executions. Participants were asked to rest for a minimum of one minute after each transfer. Additional resting time was granted to participants who requested it in order to avoid fatigue.

Accelerometer data were collected continuously for the duration of the experiment while the participant rested and moved between different scenarios. Each participant performed 12 transfers for an average of a forty-minute recording for each participant.

### Data Analysis

Descriptive statistics, were determined for demographic data of participants.

### Automatic Transfer Quality Evaluation

Following the method proposed by Hwang et al [23], the transfer assessment instrument (TAI) was reviewed to identify specific items that could be evaluated using an accelerometer. Only 3 of the 15 items listed in Part 1 of the TAI were considered (see Textbox 1). Part 2 of the TAI was excluded as the evaluator is asked to complete a series of Likert scales based on the overall evaluation of repeated transfers rather than the use of individual skills within a single transfer.

**Selected transfer assessment instrument items for the evaluation of transfers using a chest-placed accelerometer.**

Head-hip relationship (item 12)

- Subject moves the head in the opposite direction of the hips to make the transfer easier to perform

Controlled flight (item 11)

- Transfer is smooth and uses coordinated movements
- Person appears to be safe and able to complete the skill in a controlled manner

Smooth landing (item 14)

- The landing phase of the transfer is smooth and well controlled
- For example, hands are not flying off the support surface, and the subject is sitting safely on the target surface

Other evaluation items were excluded as they referred to the positioning of the wheelchair rather than the use of specific transferring skills (items 1, 2, 3, 4, 5), evaluated static body positioning rather than movement (items 6, 8, 9, 10, 13), or were only applicable to transfers performed with the assistance of a caregiver (item 15). Finally, item 7 was omitted as its clinical implications were unclear [24].

In keeping with the guidelines of the TAI 3.0, 2 trained physiotherapists, with at least four years of clinical experience and who were familiar with the use of the TAI, evaluated each transfer identified in the video by assigning a dichotomous score (ie, good or not good) for each item. Each physiotherapist evaluated the transfers independently, and any disagreements over different scores were resolved through consensus meetings.

**Figure 3:**
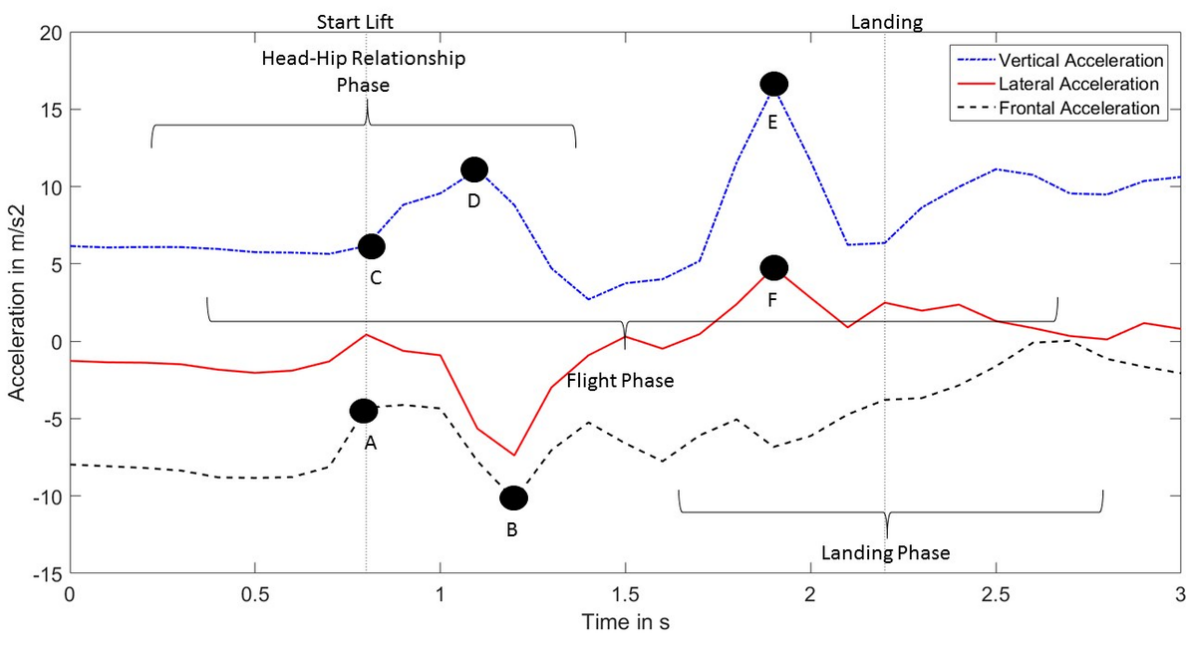
Trunk accelerations in the vertical (X), lateral (Y) and frontal (Z) direction observed during a wheelchair transfer. Vertical dotted lines mark the timestamps identified for start lift and landing used to determine time windows.

In order to segment the transfer data from the full accelerometer recording sequence, accurate timestamps for start of lift (when the buttocks of the subject lose contact with the initial surface) and landing (when the buttocks of the subject contact the target surface) were obtained from the annotated videos. The accelerometer data were then partitioned in three time windows as shown by Nawoczenski et al [25]: head-hip relationship phase, flight phase, and landing phase. Time windows were defined within a reasonable interval from the marked start and end of the transfer to accommodate for potential imprecisions due to human error when detecting start and end of the transfer. Each window corresponded to a time epoch where the selected TAI items could be evaluated (see Textbox 2, Figure 3, and Multimedia Appendix 1).

Features for head-hip relationship and landing phases were selected based on the biomechanics characteristics of wheelchair transfers and confirmed by visual inspection of the data. The rationale for the feature selection of each transfer aspect evaluated is described in the following 3 sections.

**Time epochs for automatic transfer quality evaluation.**

Head-hip relationship phase

- ±0.75s interval around the marked start lift timestamp

Flight phase

- ±0.5s interval around the marked timestamps for start lift and landing

Landing phase

- ±0.75s interval around the marked landing timestamp

#### Head-Hip Relationship

The performance of a transfer using a correct head-hip relationship requires the subject to perform a quick forward lean which causes a sharp decrease in the frontal acceleration (segment B: minimum frontal acceleration, minimum frontal jerk). A sudden trunk flexion is usually more effective in relieving weight from the buttocks (maximum total jerk). To gather more momentum, some people may move the trunk slightly backward before bending forward, leading to a greater range of frontal acceleration (segment A-B: range frontal acceleration). The direction along which the trunk moves can be represented by a line connecting the trunk with a point slightly ahead of the tip of the person’s feet. An approximation of the acceleration in that direction can be obtained from the sum of the acceleration values in the vertical direction and the inverse of the acceleration values in the frontal direction (segments A-B and C-D: maximum frontal-downward acceleration, range frontal-downward acceleration).

#### Controlled Flight

A controlled flight can be described as a smooth transition from starting to target surface, as the body follows a linear path without unexpected deviations. We, therefore, selected representative features according to preexisting literature focusing on measuring smoothness of movements during rehabilitation [26,27] (spectral length of acceleration, spectral length of velocity, dimensionless jerk, log dimensionless jerk).

#### Smooth Landing

The moment in which the subject’s buttocks land on the target surface is characterized by a sharp peak of acceleration in the vertical direction (segment E) combined with a smaller peak in the lateral direction (segment F: maximum vertical acceleration, maximum total acceleration). This would likely be reflected in higher average values of acceleration in the observed window of time (mean total acceleration, mean vertical acceleration, root mean square total acceleration). Hard landings will also likely cause large variations in the trunk accelerations, as the trunk moves to regain stability (range total acceleration).

#### Feature Selection and Modeling

The correlation-based feature subset selection method explained in Hall and Smith [28] was used to optimize the feature selection process (see Textbox 3). Only selected features were used to build the automatic transfer evaluation system. Random forest, SVM, Naïve Bayes, multinomial logistic regression (MLR) were used to build the classifiers as they are commonly used in the related literature. A leave-one-subject-out cross-validation method was used to calculate the accuracy of the models and test for generalization over unseen users.

**List of selected features calculated for automatic transfer quality evaluation.**

Head-hip relationship features

- Minimum frontal acceleration
- Range frontal acceleration
- Maximum frontal-downward acceleration
- Minimum frontal jerk

Smooth landing features

- Maximum total acceleration
- Range total acceleration
- Mean total acceleration
- Root mean square total acceleration

### Automatic Transfer Detection

Accelerometer data were divided into windows of 25 samples (1s at 25Hz) with a 50% overlap between neighboring windows. All windows were labeled for transfer occurrence according to the timestamps extracted from the videos. From each window, 59 features were extracted according to the procedure illustrated by Garcia-Masso et al [11]. Fourteen features were extracted for each accelerometer axis and the total acceleration vector including (1) SD, (2) variance, (3) 10^th^, 25^th^, 50^th^, 75^th^, and 90^th^ percentiles, (4) interquartile range, (5) range between the 10^th^ and the 90^th^ percentiles, and (6) lag-one correlation of the counts in a period of 10 seconds as a measure of temporal dynamics [11,29]. Additionally, we used a two-level wavelet transform, with Daubechies 2 as mother wavelet [11,30] to calculate the Euclidean norm of the detail coefficients of the first and second level of resolution and the approximation coefficient of the second level. Finally, we calculated sample entropy for each axis (tolerance 0.3 standard deviations, pattern length 2) as shown in [11,31] and the cross-correlation between the 3 axes.

Although wheelchair transfers were only 1 of the activities classified by Garcia-Masso et al [11] the features used were found to be very informative to discriminate between discrete types of activities undertaken by WU. Even though these activities were quite different from each other, the use of the same features would allow for the integration of transfer detection within a more general activity detection framework for the WU.

As for the automatic transfer quality evaluation, the correlation-based feature subset selection method described by Hall and Smith [28] identified 25 relevant features across all participants that were used in the classifiers’ list of attributes (see Textbox 4).

**List of selected features for the automatic transfer detection.**

Time domain features

- Variance (Z, Total)
- SD (Total)
- 10^th^ Percentile (Y, Z, Total)
- 25^th^ Percentile (Z)
- 50^th^ Percentile (Total)
- 75^th^ Percentile (Total)
- 90^th^ Percentile (Z, Total)
- Interquartile Range (X, Y, Z)
- Range between 10^th^ and 90^th^ percentiles (Y, Z, Total)
- Lag-one correlation (Z, Total)

Wavelet transform features

- Euclidean norm 1^st^ level coefficient (Y)
- Euclidean norm 2^nd^ level coefficient (Y, Z, Total)
- Approximation coefficient of the 2^nd^ level (Z, Total)

**Table 1:** Number of instances labeled according the occurrence and nonoccurrence of transfers for each participant.

Participant gender	Age (years)	Transfer (relative %)^a^	No transfer (relative %)	Total^b^
Male	26	145 (3.1%)	4520 (96.9%)	4665
Male	26	100 (2.0%)	4937 (98.0%)	5037
Male	47	105 (1.4%)	7211 (98.6%)	7316
Male	25	108 (2.6%)	4005 (97.4%)	4113
Male	30	109 (2.1%)	5219 (97.9%)	5328
Male	35	108 (2.5%)	4273 (97.5%)	4381
Male	35	101 (1.7%)	5787 (98.3%)	5888
Male	46	117 (2.2%)	5104 (97.8%)	5221
Female	58	93 (1.0%)	9022 (99.0%)	9115

^a^Refers to the ratio between instances of transfer occurrence and the instances of no transfer occurrence.

^b^Refers to the total number of instances for each participant extracted from the accelerometer data.

Only selected features were used to build the automatic transfer detection system. Classification algorithms used for transfer monitoring were the same as the one used for automatic transfer quality evaluation. A leave-one-subject-out cross-validation strategy was to evaluate the performance and generalization of the models. Having the participant wear the accelerometer for the whole duration of the experiment minimized the disruption and resulted in the collection of a more realistic dataset. Accelerometer data were recorded continuously for approximately forty minutes for each participant. However, only 12 transfers lasting for a couple of seconds each were performed within the time frame. This resulted in a severe imbalance (See Table 1) between the transfer instances (986/51064, 1.9%) and no transfer instances (50078/51064, 98.1%). To reduce classifiers bias towards the majority class, random sampling with a 1:1 transfer/no transfer ratio was used for all participants.

## Results

### Participants

Nine manual WU (8 males, 1 female) were recruited for the study. Their mean age was 36.4 years (SD 11.5), mean height was 181.5 cm (SD 13.5), and mean weight was 88.4 kg (SD 17.6). All participants were successfully able to complete the 12 transfers and no missing data were found in the dataset (see Table 2).

### Evaluation of Transfer Quality

After the physiotherapists’ evaluations, the dataset contained the following ratio of good/bad transfer instances for each evaluation item: (1) 59/49 for head-hip relationship, (2) 106/2 for controlled flight, and (3) 61/47 for smooth landing. Due to the unbalanced nature of the dataset for the controlled flight item, the automatic evaluation was not performed.

For both evaluation items, all classifiers exhibited similar average accuracies across all participants. For the evaluation of the head-hip relationship item average classifier accuracies across all participants were: (1) 75.9% (SD 13.5%) for SVM, (2) 72.2% (SD 15.6%) for random forest, (3) 75% (SD 13.8%) for Naïve Bayes, and (4) 75.9% (SD 14.1%) for MLR. For the evaluation of the smooth landing item average classifiers accuracies across all participants were: (1) 79.6% (SD 7.4%) for SVM, (2) 73.1% (SD 13.7%) for random forest, (3) 78.7% (SD 7.3%) for Naïve Bayes, and (4) 78.7% (SD 7.3%) for MLR. SVM was found to be the most accurate classifiers across all participants for the evaluation of both head-hip relationship use and smoothness of landing.

Accuracy and F1 scores displayed substantial variations across individual participants (see Table 3) while SVM classifiers achieved a balanced relative accuracy for both evaluation items (see Table 4).

### Assessment of Automatic Transfer Detection

**Figure 4:**
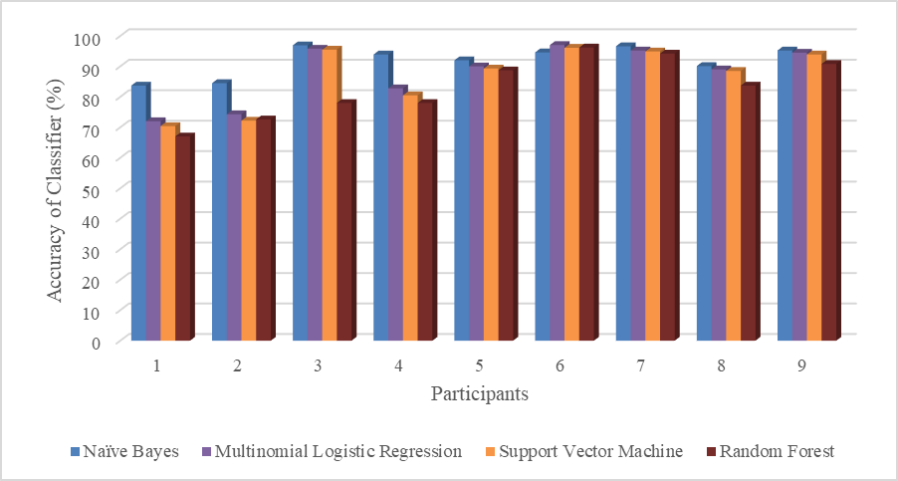
Classifiers accuracy for automatic transfer detection across all participants.

Average classifiers accuracies for automatic transfer detection were: (1) 86.8% (SD 10.1%) for SVM, (2) 83.2% (SD 10.1%) for random forest, (3) 91.9% (SD 4.9%) for Naïve Bayes, and (4) 87.8% (SD4.9%) for MLR. Overall, Naïve Bayes classifiers obtained higher classification accuracies. Naïve Bayes classifiers displayed a considerably higher relative accuracy for no transfer occurrence instances. On the other hand, MLR classifiers achieved a more balanced relative accuracy between the 2 classes (Table 5 and Figure 4).

**Table 2:** Overview of participants’ characteristics.

Participant gender	Age (years)	Medical condition	Wheelchair use (years)
Male	26	SCI^a^ C6^b^	2.1
Male	26	SCI C7	0.8
Male	47	SCI T4^c^	8.5
Male	25	SCI T5	2.8
Male	30	SCI C6	12.0
Male	35	SCI T12	3.3
Male	35	SCI T1	7.8
Male	46	SCI T5	10.9
Female	58	TM^d^	9.5

^a^SCI: spinal cord injury.

^b^C(n): Cervical spinal cord level of injury.

^c^T(n): Thoracic spinal cord level of injury.

^d^TM: transverse myelitis.

**Table 3:** Accuracy and weighted average score of support vector machine classifiers for the evaluation of head-hip relationship and smooth landing items.

Participant gender	Age (years)	SVM^a^ accuracy (head-hip relationship)	F1^b^ score	SVM accuracy (smooth landing)	F1 score
Male	26	66.7%	.667	75.0%	.739
Male	26	100.0%	1.00	83.3%	.838
Male	47	66.7%	.686	83.3%	.829
Male	25	91.7%	.923	75.0%	.755
Male	30	75.0%	.750	75.0%	.739
Male	35	66.7%	.663	66.7%	.667
Male	35	83.3%	.844	83.3%	.829
Male	46	75.0%	.767	83.3%	.833
Female	58	58.3%	.569	91.7%	.917

^a^SVM: support vector machine.

^b^F1: weighted average.

**Table 4:** Support vector machine global confusion matrices showing actual versus predicted classes (and relative percentages) for the evaluation of head-hip relationship use, and smoothness of landing for all wheelchair transfers.

Actual class	Predicted class			
	HH^a^	No HH	SL^b^	No SL
HH	31 (63.3%)	18 (36.7%)	—	—
No HH	8 (13.6%)	51 (86.4%)	—	—
SL	—	—	36 (76.6%)	11 (23.4%)
No SL	—	—	11 (18.0%)	50 (82.0%)

^a^HH: head-hip relationship.

^b^SL: smooth landing.

**Table 5:** Global confusion matrices for automatic transfer detection using Naïve Bayes and multinomial logistic regression classifiers.

Actual class		Predicted class			
		Naïve Bayes classifiers		MLR^a^	
**Naïve Bayes classifiers**		TO^b^	No TO	TO	No TO
	TO	46160 (92.8%)	3558 (7.2%)	—	—
	No TO	286 (27.5%)	754 (72.5%)	—	—
**MLR**					
	TO	—	—	44293 (89.1%)	5425 (10.9%)
	No TO	—	—	105 (15.3%)	881 (84.7%)

^a^MLR: multinomial logistic regression.

^b^TO: transfer occurrence.

## Discussion

### Principal Findings

To our knowledge, this is the first paper that has attempted to use a body-worn accelerometer to both monitor the occurrence of wheelchair transfers and evaluate their quality. Using a single body-worn accelerometer located at the chest, we were able to evaluate 2 important elements of wheelchair transfers technique: head-hip relationship use, and smoothness of landing with a respective accuracy of 75.9% and 79.9 %. These results are comparable to previous studies within the WU population, such as research which classifies wheelchair propulsion [32,33]. Unfortunately, we were unable to perform the automatic evaluation for the controlled flight item, as nearly all participants were able to control their movement during transferring. Participants in the current study were expert WU with good upper body strength. However, in a population of novice WU, this item could be particularly important in helping to identify difficulties and highlight the absence of postural control which can be linked to an increased risk of falling [34].

The choice of using a single chest-mounted accelerometer for the automatic transfer quality evaluation limited our assessment to 3 items of the TAI. However, this evaluation can have important clinical implications if extended to transfers performed in everyday settings. For example, the use of a head-hip relationship during wheelchair transfers has been shown to reduce muscular activity [35], shoulder forces [24], and increase stability [36]. Additionally, while a smooth landing is not necessarily linked with a reduction in the upper limb forces measured during wheelchair transfers, it offers an important indication of safety, as poor control in the final stage of the transfer can lead to an increased risk of falling [37].

Despite not reaching 100% accuracy, the current study shows the potential of using a single chest-mounted sensor to detect the occurrence of wheelchair transfers. Previous research by Garcia-Masso et al [11] included the chest sensor to increase the accuracy of classification when combined with wrist-mounted sensors but failed to investigate the data from the chest alone. Our results show that such a sensor is as powerful as a pair of wrist sensors in detecting transfers.

The placement on the chest also allowed for the quality of movement to be evaluated. However, this sensor alone is not sufficient to measure the exact start and end of a transfer (or other items of TAI). Therefore, future work should investigate the use of an unobtrusive second sensor to aid with accurate detection (eg, a pressure switch on the wheelchair itself).

It should be noted that, even if the data from the current study are not directly comparable with [11], our dataset had increased complexity due to its higher ecological variability and to the continuous detection of such events. Indeed, we attempted to replicate a typical pattern of daily activities within a WU’s day by asking participants to wear the accelerometer while traveling and resting between scenario activities. This makes detecting transfers a more difficult task than when transfers are completed cyclically for up to a minute at a time between surfaces of a fixed height, and without any change in scenario.

The detection of transfers was more successful for some participants than others. The Naïve Bayes classifiers were the most accurate across all participants. However, it was unbalanced and overpredicted the number of transfers when no transfer was present. Despite this the Naïve Bayes classifiers were more robust, ensuring an accuracy of more than 80% for all participants. When the more balanced MLR was used the accuracy for participant 1 (male, 26 years of age) and 2 (male, 26 years of age) dropped below 70%. It is unclear why these participants were so affected. Future work should look to replicate our work in the wild and with a larger and more heterogeneous sample of WU, which we believe would begin to address the limitations of the current dataset. In fact, despite our efforts, the current set of participants included mainly males with SCI. Although the imbalance of genders and medical conditions among participants are not uncommon in wheelchair studies [38], it can limit the potential for generalization of results. Further research could also be carried out to identify alternative and additional locations for sensor positioning with the aim to maximize the accuracy of transfer detection.

### Future Developments

Overall, the use of machine learning techniques to automatically detect and evaluate wheelchair transfers shows good potential for future clinical and well-being applications. A wearable system would allow people to self-monitor their transfers and seek additional medical help as and when required. Also, the system could be used to provide feedback to WU, helping them to identify potential weaknesses and providing suggestions for improvements. If paired with data concerning, for example—the environment, the emotional state of the WU, and time of day—a more complex picture of wheelchair transfers can be built, and better feedback and support mechanisms put in place. Therefore, the larger aim of our project is to develop a wearable system capable of continuously tracking and giving real-time feedback to WU on their transfer ability as they go about their life. Future developments should look into the possibility to combine the chest accelerometer with a portable surface electromyography system placed on the arm, as this could allow for a complete picture of the transfer skills to be captured. This information could then be used to provide more detailed feedback to the WU to help them train and practice the movement in real-life contexts.

Finally, the ability to easily map transfer difficulties in the built environment could also allow WU to share their experiences and provide information about accessibility standards of various establishments (ie, hotel rooms, restaurant toilet). This could also be extended to lower- and middle-income countries, where the majority of disabled people live, who frequently do not have access to rehabilitation programs [39].

### Conclusions

In this study, we investigated the use of a single chest-mounted accelerometer to monitor the occurrence of wheelchair transfers and evaluate their quality under three ecological settings. Using features extracted from the accelerometer we were able to improve the accuracy of detection of transfers for the ubiquitous computing literature in this area while also detecting key elements of the quality of movement at performance levels observed for other aspects of the movements. Results from this study open new possibilities for unobtrusive monitoring and evaluation of the performance of wheelchair transfers in the real world that could lead to important applications for wheelchair transfers training, upper limb injury prevention, and improved accessibility.

## Appendix

Multimedia Appendix 1 Clinical evaluation of wheelchair transfers.

## Abbreviations

C(n): cervical spinal cord level of injury
F1: weighted average
HH: head-hip relationship
MLR: multinomial logistic regression
QDA: quadratic discriminant analysis
SCI: spinal cord injury
SL: smooth landing
SVM: support vector machine
TAI: transfer assessment instrument
TM: transverse myelitis
TO: transfer occurrence
T(n): thoracic spinal cord level of injury
WU: wheelchair user(s)
