# Supplementary material for: Use of a Low-Cost, Chest-Mounted Accelerometer to Evaluate Transfer Skills of Wheelchair Users During Everyday Activities: Observational Study
Source: JMIR Rehabil Assist Technol. 2018 Dec 20;5(2):e11748. doi: 10.2196/11748 (PMC6320409; doi:10.2196/11748)
Supplement: Multimedia Appendix 1 [file rehab_v5i2e11748_app1.pdf]

Multimedia Appendix 1

Clinical evaluation of wheelchair transfers

The challenge of evaluating wheelchair transfers lies in being able to detect the quality of movement, something hitherto only been possible through clinical evaluation. Therefore, understanding the clinical process is important for understanding the selection of features. Wheelchair transfers have a crucial role in the everyday life of many wheelchair users. Wheelchair skills tests, which structure and inform rehabilitation programmes include at least one item that measures an individual's ability to transfer [37]. Despite this, in most clinical settings the evaluation of transfer performance is still mainly based on unstructured visual assessment conducted by physiotherapists and occupational therapists [38]. The most common type of transfer is an unassisted sitting transfer, which is now described and is the focus of this paper. This transfer is used by people unable to weight bear through the lower limb, such as people who have suffered a spinal cord injury.

To perform an unassisted sitting transfer the person will first position their wheelchair close to the surface on which he/she wishes to transfer to. Usually the chair will be placed at a slight angle from the target surface and the person will scoot towards the front of the seat. Some users will place both feet on the floor while others might prefer to leave them on the footplate. The person will then place one hand (the leading hand) close to the target surface and will then leave one hand (the trailing hand) on the wheelchair. The transfer itself is usually achieved with a quick forward lean of the trunk. This releases weight from the buttocks and is accompanied by a pivoting motion that rotates the trunk so that the shoulders point in the opposite direction to the direction of travel of the transfer. This motion might seem counter intuitive; however, it is critical to a successful transfer. This 'head-hip relationship' as it is called, means the head will move in the opposite direction to the hips during the transfer. The sequence of events for an unassisted sitting transfer is shown in Figure 5.

The Transfer Assessment Instrument (TAI) is a validated clinical scale used to evaluate the quality of sitting and standing wheelchair transfers performed by wheelchair users [18]. The TAI was refined by [16] in order to improve reliability and validity. This improved version (version 3.0) features two parts. Part 1 is composed of 15 items. Each item corresponds to a small component of the transfer such as the placement of a hand or the feet, or the smoothness of movement. Clinicians score each part of the transfer using a dichotomous scale. Part 2 provides an overall evaluation of repeated transfers using 12 items rated on a 4-point Likert scale. Areas of evaluation focus on transfer set-up, quality and the implementation of strategies to prevent upper limb injuries. Although the TAI is reliable, especially when used by people who have received appropriate training, it is only very recently that links between the TAI and the kinetics of the limbs' movement (using high cost sensors) have been established in biomechanical lab settings [23]. We aim to extend this work to be able to transform aspects of the TAI into low-cost sensors measurements that can be brought into real-life contexts and integrated into coaching apps.

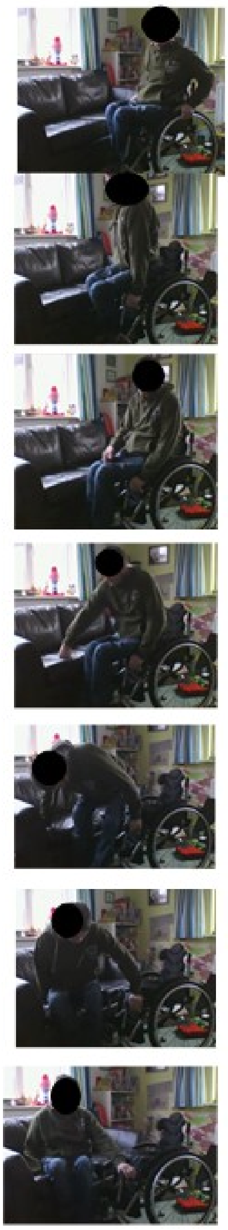

Wheelchair positioned beside the target seat

Forward scoot

Feet moved on the floor

Hands positioned on starting and target surface

Start of the lift phase (seat-off)

Landing

Transfer complete

Figure 5 Sequence of movements used to perform an unassisted sitting transfer
